# Vasoreactivity in CADASIL: Comparison to structural MRI and neuropsychology

**DOI:** 10.1177/0271678X17710375

**Published:** 2017-05-24

**Authors:** Fiona C Moreton, Breda Cullen, Christian Delles, Celestine Santosh, Rosario L Gonzalez, Krishna Dani, Keith W Muir

**Affiliations:** 1 Institute of Neuroscience and Psychology, University of Glasgow, Queen Elizabeth University Hospital, Glasgow, UK; 2 Institute of Health and Wellbeing, University of Glasgow, Queen Elizabeth University Hospital, Glasgow, UK; 3 Institute of Cardiovascular and Medical Sciences, University of Glasgow, Glasgow, UK; 4 Department of Neuroradiology, Institute of Neurological Sciences, Queen Elizabeth University Hospital, Glasgow, UK; 5 Department of Clinical Physics and Bioengineering, Glasgow Royal Infirmary, Glasgow, UK

**Keywords:** MRI, ASL, lacunar infarcts, genetics, stroke

## Abstract

Impaired cerebrovascular reactivity precedes histological and clinical evidence of CADASIL in animal models. We aimed to more fully characterise peripheral and cerebral vascular function and reactivity in a cohort of adult CADASIL patients, and explore the associations of these with conventional clinical, imaging and neuropsychological measures. A total of 22 adults with CADASIL gave informed consent to participate in an exploratory study of vascular function in CADASIL. Clinical assessment, comprehensive vascular assessment, MRI and neuropsychological testing were conducted. We measured cerebral vasoreactivity with transcranial Doppler and arterial spin labelling MRI with hypercapnia challenge. Number and volume of lacunes, subcortical hyperintensity volume, microbleeds and normalised brain volume were assessed on MRI. Analysis was exploratory and examined the associations between different markers. Cerebrovascular reactivity measured by ASL correlated with peripheral vasoreactivity measured by flow mediated dilatation. Subjects with ≥5 lacunes were older, with higher carotid intima-media thickness and had impaired cerebral and peripheral vasoreactivity. Subjects with depressive symptoms, disability or delayed processing speed also showed a trend to impaired vasoreactivity. Impaired vasoreactivity and vascular dysfunction may play a significant role in the pathophysiology of CADASIL, and vascular assessments may be useful biomarkers of severity in both longitudinal and clinical trials.

## Introduction

Cerebral autosomal dominant arteriopathy with subcortical infarcts and leukoencephalopathy (CADASIL) is the most common monogenetic small vessel disease and results in stroke and cognitive impairment.^1^ CADASIL is caused by mutations of *NOTCH3*, which codes for a receptor expressed in vascular smooth muscle cells and pericytes,^2^ involved in regulation of cerebral blood flow (CBF).^3^ Although clinical manifestations are confined to the brain, arteriopathy is detected throughout the body.^4^

Coincident with histological abnormalities, vascular dysfunction occurs. Human studies suggest reduced CBF, which may precede symptoms,^5^ and impairment of cerebrovascular reactivity (CVR) within white matter hyperintensities on brain magnetic resonance imaging (MRI).^6^ Impaired CVR was associated with increased hyperintensity volume after seven years, but the clinical relevance of this remains unclear.^7^ Peripheral vascular function may also be impaired in vivo,^8^ and ex vivo*.*^9^ Other measures of vascular disease such as carotid intima-media thickness^10^ and blood pressure (BP)^11^ may influence disease severity.

Genetic testing, and the long presymptomatic phase, potentially allow for early intervention. MRI demonstrates features of small vessel disease, including hyperintensities, lacunes, microbleeds and atrophy. Whilst lacunes and atrophy are associated with cognitive deterioration,^12^ the wide inter-individual variation and slow rate of progression potentially limit the utility of structural MRI features as biomarkers of disease progression.^13^ Vascular function may offer an alternative index of disease activity and progression.

We aimed to explore cerebral and peripheral vascular function and reactivity in a cohort of adult CADASIL patients to give a more complete view of this than performed in previous studies. We investigated associations with structural MRI, clinical and neuropsychological markers of disease, and discuss the role for potential biomarkers in clinical trials.

## Material and methods

### Study cohort

Subjects aged 18 years or over with a genetic diagnosis of CADASIL were eligible. Exclusion criteria included contraindications to MRI, current use of calcium channel blockers or angiotensin-converting enzyme inhibitors, and type II respiratory failure. Subjects provided written, informed consent. The study was approved by the West of Scotland Research Ethics Service (12/WS/0295). The study was governed by standards laid down in the Helsinki Declaration of 1975 (and as revised in 1983).

Study visits were: (1) Transcranial Doppler (TCD) ultrasound and clinical assessment, (2) peripheral vascular tests, (3) MRI with respiratory challenge, and (4) neuropsychology. Visit 1 always took place first but otherwise visits took place in any order. Subjects were instructed not to take caffeine, nicotine or alcohol for 4 h prior to visits 1, 2 and 3.

### Visit 1: Clinical assessment and TCD ultrasound

Details regarding medical history, medication and cardiovascular risk factors were collected. Neurological impairment was assessed using the National Institute of Health Stroke Scale (NIHSS).^14^ Global disability was assessed with the Rankin Focused Assessment tool to derive the categorisation on the modified Rankin Scale (mRS).^15,16^ Concurrent presence of anxiety or depressive symptoms was recorded with the self-rating Hospital Anxiety and Depression Scale (HADS, GL Assessment Limited, London).^17^

TCD of the middle cerebral arteries in the transtemporal window was performed with a ST3/Model PMD 150 (Spencer Technologies, Seattle, USA) with continuous monitoring (IntelliVue MP30, Philips Medical Systems, the Netherlands) including oxygen saturation and end-tidal CO_2_ (EtCO_2_). BP was measured before, during and after CO_2_ delivery. An anaesthetic mask (Quadralite mask, ref 7193, Intersurgical Ltd) attached with a harness (Ref 2224, Intersurgical Ltd) was fitted to the patient, and attached to a unidirectional breathing circuit (Ref 2013014, Intersurgical Ltd). Mean flow velocity (MFV; cm/s) was recorded continuously whilst the subject received: 3 min room air – 3 min 6% CO_2_/air at 40 L/min (BOC Medical, Manchester, UK, Medical Special’s Licence Number ML/0735/01) – 3 min room air. Traces were excluded if EtCO_2_ was not maintained during the hypercapnia challenge suggesting a circuit leak. If both right and left measurements were available, the mean was used. Cerebrovascular reactivity (CVR_TCD_) was calculated as a percentage change in MFV per change in EtCO_2_.

$${\text{CVR}}_{\text{TCD}}=\frac{{\text{MFV}}_{\text{hypercapnia}}-{\text{MFV}}_{\text{normocapnia}}}{{\text{MFV}}_{\text{normocapnia}}}\times \frac{100}{{\text{EtCO}}_{2\text{hypercapnia}}-{\text{EtCO}}_{2\text{normocapnia}}}$$

### Visit 2: Peripheral vascular tests

**Figure 1.**
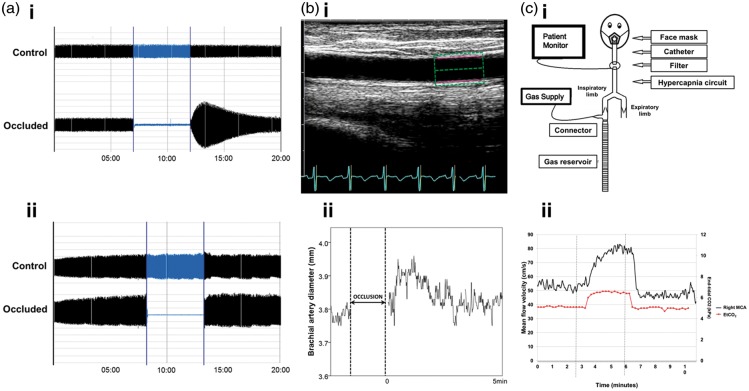
Assessing peripheral and cerebral vasoreactivity. (a) Endothelium-dependent vasodilatation was assessed using a plethysmographic method (EndoPat-2000). Following occlusion, hyperaemia should occur if there is normal endothelial function (ai), whereas absence of hyperaemia suggests endothelial dysfunction (aii). (bi) Endothelial function was also assessed with ultrasonography of the brachial artery with change in diameter being measured with automated identification of the intima-media layer (green and pink box) (bii). Following distal artery occlusion, a flow-mediated dilatation (FMD) of the brachial artery should occur, with a gradual return to baseline, and an example of this is shown. (ci) Cerebral reactivity challenges were performed using a hypercapnic challenge delivered with a unidirectional circuit and mask. (cii) Transcranial Doppler ultrasound assessed mean flow velocity (MFV) over a 9-min test period, with 3 min of 6% CO_2_. Cerebrovascular reactivity was calculated as the change in velocity between the last minute of hypercapnia and the baseline. Flow in the right middle cerebral artery (MCA; black line) and end-tidal CO_2_ (red line) are shown.

All peripheral vascular tests took place within a temperature controlled room (22–24℃) and were performed and analysed by a single rater (FM). Body mass index (BMI) was calculated. Supine BP and heart rate were measured following 10 min of rest. Pulse wave analysis (PWA, as the augmentation index at 75 beats per minute, AI75) and pulse wave velocity (PWV) assessed large vessel arterial stiffness; carotid ultrasound with carotid intima-media thickness (CIMT) assessed generalised atherosclerosis; and non-invasive peripheral artery tonometry (reactive hyperaemia index, RHI) and flow mediated dilatation (FMD) of the brachial artery assessed endothelial function (see Figure 1 for example). Further details and background about methods are shown in supplementary materials.

### Visit 3: MRI

Scans were obtained on a 3T MRI GE Signa Excite HD scanner with an 8-channel head coil (GE Medical Systems, Milwaukee, Wisconsin). Sequences included (i) axial T2-weighted FLAIR (repetition time/inversion time/echo time = 10,000/2250/140 ms, slice thickness = 5 mm, interslice gap = 1.5 mm, matrix 384 × 256, acquisition time = 3:20 min); (ii) axial T1-weighted FSPGR BRAVO (repetition time/inversion time/echo time = 9.0/450/3.6 ms, slice thickness = 1 mm, no interslice gap, flip angle = 12°, matrix 320 × 320, acquisition time = 4:28 min); (iii) axial T2*-weighted susceptibility angiography (SWAN) (repetition time/echo time = 40/25 ms, slice thickness 3.6 mm, slice gap = 1.8 mm, flip angle 15°, matrix 320 × 224, acquisition time = 2:16 min); (iv) sagittal Inhance 3D velocity MR angiography (repetition time/echo time = 8.6/3.54 ms, slice thickness = 1.2 mm, flip angle = 8°, matrix 320 × 224, acquisition time = 3:11 min). Diffusion tensor imaging and resting state BOLD scans were also obtained but not analysed in this study.

All Arterial Spin Labeling (ASL) scans took place between 9:30 am and 13:45 pm. Subjects were fitted with a close-fitting mask (Intersurgical Ltd, Ref 1141). Expiratory ports were closed and gauze swabs and tape were used to improve fit. The mask was connected to the unidirectional breathing circuit (Ref 2013014, Intersurgical Ltd). Observations (oxygen saturation, respiratory rate, heart rate) and gas concentrations were measured with an MRI compatible monitor (Veris® Vital Signs Monitor, MEDRAD, Indianola, USA). A 3D pseudo-continuous ASL scan was performed with the subject receiving 15 L/min air. The subject was then switched to receive 40 L/min of 6%CO_2_/air. After 2 min, the ASL scan was repeated. The protocol was as follows: repetition time = 4864 ms, echo time = 10.1 ms, labelling duration = 1500 ms, post-labelling delay time = 2025 ms, slice thickness 3.5 mm, matrix 128 × 128, flip angle 155°, NEX 3.0, time = 4.42 min. In the first three patients, a neurovascular head coil (eight-channel) was used when obtaining the ASL scans.

### MRI analysis

Cerebral microbleeds, lacunes and subcortical hyperintensities (SHs) were defined as per recent neuroimaging standards.^18^ The Microbleed Anatomical Rating Scale (MARS), a validated scale, was used to classify microbleeds.^19^ One rater (FM) scored all scans twice: for any scans where there was discrepancy, a second person reviewed the scan (KD). Lacunes were defined as subcortical, fluid-filled cavities (signal similar to cerebrospinal fluid) of between 3 mm and 15 mm in diameter.^18^ Lacunes were segmented and their volume calculated on T1-weighted images using 2D and 3D thresholding tools (Analyze v 11.0, AnalyzeDirect Inc., USA). Lacune volume was normalised (NLV, %) to intracranial cavity volume. Intraclass correlation for this method for 20 scans was 0.934 (0.845–0.973).

FMRIB software library v 5.0 tools including SIENAX, were used to determine intracranial cavity volume from SWAN images and normalised brain volume (NBV) from T1-weighted images.^20–22^ Hyperintense signal abnormalities on FLAIR in white matter, grey matter and brainstem were termed SHs.^18^ To calculate the SH volume, a skull stripped FLAIR image was created^20,21,23^ and the mode intensity of this image, multiplied by 1.3, was used to threshold the image, with manual removal of cortical voxels if required^24^ to create an SH mask. SH volume was normalised (NSH, %) by intracranial cavity volume. Intraclass correlation for five scans repeated by one rater with this method was 0.999 (0.991 − 1).

MR angiography was inspected by a neuroradiologist (CS) for evidence of vessel stenosis.

### ASL

**Figure 2.**
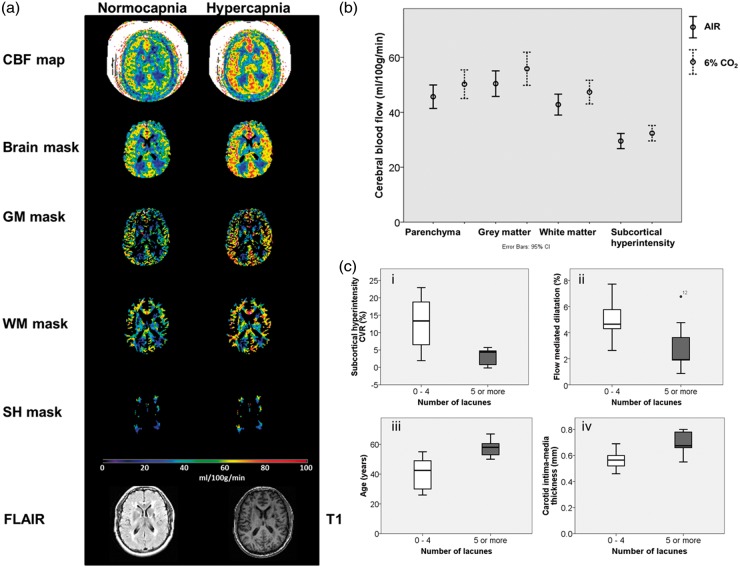
Cerebral blood flow and vasoreactivity – ASL MRI. (a) Generated CBF maps were masked with brain, grey matter, white matter and subcortical hyperintensity masks and the average in each mask recorded. Masks were created using T1 and FLAIR images. Scale bar shown for CBF in ml/100 g/min (b) CBF in brain, grey matter, white matter and subcortical hyperintensities whilst breathing air (line) and 6% CO_2_ (dashed line). (c) Patients with five or more lacunes had (i) lower cerebral vasoreactivity and (ii) lower peripheral vasoreactivity (brachial FMD). There were also (iii) older and had higher carotid intima-media thickness (iv). Boxplot shows medians, quartiles, and extreme values. CBF: cerebral blood flow; GM: grey matter; WM: white matter; SH: subcortical hyperintensity.

Structural scans, SH masks and ASL scans were co-registered (Analyze v 11). Quantitative CBF maps were generated using an in house macro for Image J (Rasband, W.S., ImageJ, U. S. National Institutes of Health, Bethesda, Maryland, USA, http://imagej.nih.gov/ij/, 1997–2014.) The transformed T1-weighted image was segmented into parenchyma, grey, and white matter masks.^23^ SH pixels were removed from grey and white matter masks. Masks were applied to CBF maps (see Figure 2).

Change in CBF (%ΔCBF) was corrected for change in EtCO_2_ to calculate cerebrovascular reactivity (CVR_ASL_)

$${\text{CVR}}_{\text{ASL}}=\frac{{\text{CBF}}_{\text{hypercapnia}}-{\text{CBF}}_{\text{normocapnia}}}{{\text{CBF}}_{\text{normocapnia}}}\times \frac{100}{{\text{EtCO}}_{2\text{hypercapnia}}-{\text{EtCO}}_{2\text{normocapnia}}}$$

Expiratory gas data were used if EtCO_2_ whilst breathing air was between 3.5 and 6.5%, suggesting reasonable mask seal.

### Visit 4: Neuropsychological assessment

Neuropsychological testing was conducted by a specialist clinical neuropsychologist (BC). Assessment focussed on processing speed and executive function as the primary cognitive domains affected in CADASIL. Composite scores were calculated as the mean of the domain-specific individual tests after conversion of raw scores to standardised scores (z-scores, corrected for age or age and education, with reference to published normative tables). The processing speed composite score was calculated from the Symbol-Digit Modalities test and Trail-making test Part A. The executive function composite score was calculated from the Similarities sub-test of the Wechsler Adult Intelligence Scale, the FAS letter fluency test, the Trail-making Test Part B and the Stroop Neuropsychological Screening Test. For both composite scores, a higher score indicates better performance. Subjects included in the analysis had no known visual disabilities that would impair performance, but one subject was unable to complete some tests due to dysarthria and inability to hold a pen.

### Statistical analysis

Statistical analysis was performed with IBM SPSS Version 21 (IBM Corp, Armonk, NY, USA). This was an exploratory study so multiple variables were included and compared. Variables investigated included age, systolic BP, PWV, FMD, CIMT, RHI, CVR_TCD_, CBF and CVR_ASL_ in different brain regions. Clinical outcomes were: history of stroke, processing speed and executive function. Outcomes of clinical scales were dichotomised: NIHSS (0 or ≥1), mRS (0–1 or ≥2) and HADS depression and anxiety scores (0–7 or ≥8)

Continuous variables were compared with Spearman’s rank correlation. Regional CBF was compared with paired t-tests. Structural MRI variables were dichotomised by their median. Normality was tested with Shapiro-Wilk. For categorical outcomes, normally distributed data were tested with independent t-test, and non-normally distributed data with independent sample Mann Whitney U tests. Results were expressed as mean (standard deviation, SD) unless otherwise stated. Although multiple comparisons were used, this was an exploratory study so significance was set at p < 0.05. Multivariate testing was not undertaken due to small sample size.

## Results

A total of 22 subjects from 19 pedigrees were recruited. There were nine different mutations, in five different exons. Subject demographics, risk factors, history and imaging characteristics are reported in Table 1. All subjects attended all study visits over a mean of 79 days (standard deviation 26 days). There was no evidence of extracranial vessel disease on MRA in 20/22 (one subject aged 30 years did not undergo MRA due to technical problems; one scan had movement artefact, but the patient had normal carotid ultrasound).

**Table 1.** Study cohort characteristics (n = 22).

Characteristics	Cohort (n = 22)	
Demographic characteristics		
Age, mean (SD) (years)	49.6	(11.2)
Female, n (%)	11	(50)
Clinical scores		
NIH stroke scale, median (range)	0	(0–3)
Modified Rankin Score, median (range)	0	(0–3)
Anxiety score, median (range)	6	(2–16)
Depression score, median (range)	4	(0–18)
Clinical features, n (%)		
Stroke or TIA	11	(50)
Migraine	21	(95)
Depression	10	(45)
Urinary incontinence	4	(18)
Seizures	0	(0)
Vascular risk factors, n (%)		
Current or ex-smoker	11	(50)
Hypertension^a^	0	(0)
Hypercholesterolaemia^b^	13	(59)
Diabetes mellitus	0	(0)
Medication, n (%)		
Statin	16	(73)
Antiplatelet	18	(82)
Antidepressant	8	(36)
Beta-blocker^c^	2	(9)
Imaging characteristics, mean ± SD, range, median^d^		
No. of lacunes	9 ± 10, 0–34, 5	
No. of microbleeds	2 ± 3, 0–10, 0	
Normalised lacune volume (NLV), %	0.04 ± 0.04, 0–0.15, 0.02	
Normalised SH volume (NSH), %	6.0 ± 3.9, 1.0–15.5, 5.2	
Normalised brain volume (NBV), L	1.5 ± 0.1, 1.4–1.8, 1.5	

SD: standard deviation; NIH: National Institute of Health; TIA: Transient Ischaemic Attack.

a Defined as systolic BP > 140 or diastolic BP > 90 mmHg on two or more occasions, or reported in patients’ medical records.

b Defined as a recorded reading of cholesterol >5.2 mmol/L.

c Prescribed for migraine prevention rather than blood pressure control.

d T1 and FLAIR movement artefact in one subject; two subjects were excluded from microbleed assessment due to incorrect slice thickness.

CBF measured by ASL was available in 19 subjects (one not performed, two excess head rotation prevented analysis). CVR_ASL_ was available in 13 patients. Study cohort vascular measurements are shown in Table 2.

**Table 2.** Study cohort vascular measurements.

	Number	Mean (SD)	
SBP, mmHg	22	120	(11)
PWA, augmentation index at 75 bpm	22	17	(13)
PWV, m/s	21	7.5	(1.1)
RHI, %	20	2.1	(0.7)
CIMT, mm	21	0.64	(0.1)
FMD, %	18	4.1	(1.9)
MFV, cm/s	21	40	(9.5)
Brain parenchyma CBF, ml/100 g/min	19	46	(8.6)
Grey matter CBF, ml/100 g/min	19	51	(9.4)
White matter CBF, ml/100 g/min	19	43	(7.6)
Subcortical hyperintensity CBF, ml/100 g/min	19	30	(5.6)
CVR_TCD_, %	21	19	(11.2)
Grey matter CVR_ASL_, %	13	9.5	(9.3)
Subcortical hyperintensity CVR_ASL_, %	13	9.0	(7.7)

SBP: systolic blood pressure; BMI: body mass index; PWA: pulse wave analysis; PWV: pulse wave velocity; RHI: reactive hyperaemia index; CIMT: carotid intima-media thickness; FMD: flow mediated dilatation; MFV: mean flow velocity; CBF: cerebral blood flow.

### Blood flow and reactivity

CBF was highest in grey matter compared to white matter (p < 0.001) or SHs (p < 0.001; Table 2). CBF and MFV were not significantly correlated (grey matter CBF, n = 18, r_s_ = 0.320, p = 0.195), or related to age (grey matter CBF, n = 19, r_s_ = −0.383, p = 0.106; MFV, n = 21, r_s_ = −0.370, p = 0.099). Hypercapnic-induced changes in blood flow varied widely among patients but, intra-subject measures in individual brain regions were highly correlated with each other (r_s_ values between 0.940 and 0.995).

Grey matter CVR_ASL_ showed a non-significant trend towards association with CVR_TCD_ (n = 13, r_s_ = 0.484, p = 0.094). FMD was positively correlated with CVR_ASL_ (parenchyma, n = 12, r_s_ = 0.615, p = 0.033; grey matter, n = 12, r_s_ = 0.566, p = 0.055).

Resting systolic BP was positively correlated with all measures of brain reactivity (grey matter CVR_ASL_, n = 13, r_s_ = 0.567, p = 0.043) and CVR_TCD_ (n = 21, r_s_ = 0.462, p = 0.035). BP did increase during hypercapnia TCD challenge (baseline 119 ± 11; max during hypercapnia 127 ± 12 mmHg, p < 0.01) but change in BP did not correlate with reactivity measures.

Measures of peripheral vasoreactivity, brachial FMD and RHI, did not correlate with each other (n = 17, r_s_ = −0.184, p = 0.479).

Current smokers or those with a greater than 20 year pack history, had higher RHI than those who had never smoked (1.9 ± 0.5 v 2.6 ± 0.8; p = 0.032). Subjects not on statins tended to be younger (42 years ± 13 v 52 ± 9; p = 0.049) but other vascular measures did not vary.

### Is peripheral and cerebral vessel function associated with structural MRI markers?

Associations between structural MRI markers and vascular measures are shown in Table 3. A lower NBV was associated with increased PWV and CIMT, and lower CVR. The presence of many lacunes (≥5) was associated with higher age, increased CIMT, lower FMD and lower CVR (Figure 2). There was a trend towards patients with microbleeds being older (absence 46yrs (11); presence 53 (11); p = 0.08), but otherwise they were not related to other vascular variables. Normalised SH volume was not significantly associated to any vascular variable. Gender and smoking history had no effect on structural MRI markers.

**Table 3.** Structural MRI markers compared to vascular measures.

		No. of lacunes		Normalised brain volume (L)		Normalised lacune volume (%)		Normalised subcortical hyperintensity volume (%)		Number of microbleeds	
		0–4	>5	**<1.548**	≥**1.548**	**<0.024**	≥**0.024**	<5.23	>5.23	**Absent**	Present
Age	n Mean (SD)	10 41 (10)	11 57 (5)	11 46 (11)	10 53 (12)	10 44 (11)	11 55 (10)	10 46 (14)	11 53 (7)	11 46 (11)	9 53 (11)
	p value	**<0.001**			0.191		**0.016^a^**		0.165		0.080**^a^**
SBP	n Mean (SD) p value	10 122 (11)	11 118 (11) 0.354	10 122 (12)	11 118 (10) 0.371	12 122 (11)	9 118 (12) 0.429	10 122 (12)	11 118 (11) 0.490	11 121 (10)	9 120 (12) 0.874
PWA	n Mean (SD) p value	10 15 (15)	11 20 (12) 0.402	11 16 (16)	10 19 (11) 0.713	10 17 (14)	11 18 (13) 0.916	10 14 (2)	11 20 (12) 0.264	11 14 (14)	9 19 (12) 0.408
PWV	n Mean (SD) p value	10 7.2 (0.9)	10 7.9 (1.1)	11 7.0 (0.9)	9 8.0 (0.8)	10 7.2 (1.0)	11 7.9 (1.1)	10 7.4 (1.0)	10 7.7 (1.1)	11 7.4 (1.2)	8 7.8 (1.1)
		0.109			**0.01**		0.197		0.540		0.513
CIMT	n Mean (SD) p value	10 0.57(0.1)	10 0.70(0.8)	10 0.59(0.1)	9 0.69(0.1)	10 0.60(0.1)	10 0.67(0.1)	9 0.61(0.1)	0.66(0.1)	11 0.63(0.1)	9 0.65(0.1)
		**0.001**			**0.025**		0.170		0.260		0.547
RHI	N Mean (SD) p value	10 1.9 (0.5)	9 2.4 (0.7)	10 2.0 (0.6)	9 2.3 (0.8)	10 1.9 (0.5)	9 2.5 (0.7)	8 2.3 (0.7)	11 2.1 (0.7)	10 2.2 (0.7)	9 2.3 (0.6)
		0.080			0.549**^a^**		**0.035**		0.476		0.739
FMD	N Mean (SD) p value	10 4.9 (1.4)	7 2.9 (2.1)	11 4.5 (1.7)	6 3.3 (2.2)	9 4.9 (1.4)	8 3.1 (2.0)	9 4.1 (1.8)	8 4.1 (2.2)	11 4.1 (1.4)	6 3.4 (2.1)
		**0.034**			0.218		**0.044**		0.991		0.396
TCD CVR	n Mean (SD) p value	10 21 (13)	10 19 (10) 0.728	11 20 (12)	9 20 (12) 0.910	10 21 (12)	10 19 (11) 0.776	9 21 (14)	11 19 (10) 0.624	10 17 (12)	9 21 (11) 0.431
GM CBF	n Mean (SD) p value	10 55 (9)	9 47 (8) 0.065	10 55 (9)	9 47 (8) 0.076	10 53 (9)	9 39 (10) 0.334	10 53 (8)	9 49 (10) 0.418	10 52 (8)	7 47 (9) 0.235
GM CVR	n Mean (SD) p value	8 14 (9)	5 2 (4)	8 13 (8)	5 3 (3)	7 12 (7)	6 6 (6)	7 11 (11)	6 8 (7)	7 11 (10)	4 8 (7)
		**0.013**		**0.021**		0.181		0.644		0.609
SH CVR	n Mean (SD) p value	**8 13 (8)**	**5 3 (3) 0.008**	8 13 (8)	**5 3 (3) 0.009**	7 11 (7)	6 6 (8) 0.280	7 11 (9)	6 7 (5) 0.372	7 11 (9)	4 8 (5) 0.528

SBP: systolic blood pressure; PWA: pulse wave analysis (augmentation index at 75bpm); PWV: pulse wave velocity; CIMT: carotid intima-media thickness; RHI: reactive hyperaemia index; FMD: flow mediated dilatation; TCD: transcranial Doppler ultrasound; CVR: cerebrovascular reactivity; GM: grey matter; CBF: cerebral blood flow; SH: subcortical hyperintensity.

a Non-parametric test used.

### Investigate how vasoreactivity relates to clinical and neuropsychological markers of disease

Subjects with depressive symptoms (HADS ≥ 8) showed a non-significant trend towards reduced CVR_ASL_ but this did not reach statistical significance (HADS ≥ 8, n = 3, grey matter CVR _ASL_ 1 (5); HADS < 8, grey matter CVR_ASL_ 12 (9); p = 0.086). Disabled patients (mRS ≥ 2) showed similar results, but all also had high depressive symptoms. Vascular measures did not vary with NIHSS or anxiety scores.

Processing speed declined with high NLV (n = 20, r_s_ = −0.667, p = 0.001) and lower NBV (n = 20, r_s_ = 0.512, p = 0.021). Impaired processing speed was associated with higher CIMT (n = 20, r_s_ = −0.463, p = 0.04) and non-significantly with lower CVR_ASL_ (grey matter n = 13, r_s_ = 0.50, p = 0.082). Impaired executive function was non-significantly associated with a lower NBV (n = 19, r_s_ = 0.441, p = 0.059) but no other measures. See supplementary materials for full results.

## Discussion

In this exploratory study, we comprehensively evaluated multimodal indices of cerebral and peripheral vascular function and investigated how these correlate to clinical and radiological features in CADASIL.

As far as we are aware, this is the first study using ASL in CADASIL, and we have demonstrated its feasibility and potential utility. Measured grey matter CBF of 51 ml/100 g/min is consistent with the literature,^25^ and regional differences were detectable, with the technique offering the advantages of repeatability, quantification and no need for contrast administration. Combining ASL with a hypercapnia challenge, again novel in this disease, we showed impaired cerebral vasoreactivity, was related to number of lacunes and brain atrophy, important correlates of clinical impairment.^26^ Patients with greater disability, depression and impaired processing speed, also tended to have worse cerebral vasoreactivity.

Peripheral vasoreactivity was also impaired in patients with higher numbers of lacunes. Whether impaired cerebral vasoreactivity pre-exists, or is subsequent to, the development of lacunes cannot be answered in this cross-sectional study. The finding of co-existent peripheral vasoreactivity does suggest, however, that abnormal vessel responsiveness in CADASIL is not solely secondary to brain damage, and may thus represent a primary process in disease evolution. Peripheral vascular testing is easier to undertake, particularly in disabled individuals, and tests have been used as biomarkers in cardiovascular studies.^27,28^ It could therefore be considered as a useful adjunct in clinical trials. Previous studies have not demonstrated differences in FMD between CADASIL patients and controls^8,29^ whereas we found it to be associated with lacunes. We measured vessel diameter continuously for 5 min after cuff deflation, rather than at discrete time points,^29^ allowing us to characterise vessel response more accurately. However, RHI was higher in patients with more lacunes, which would not be expected. This may be as RHI and FMD are not measuring the same vascular parameter, or that changes in RHI are related to shape of the response to hyperaemia rather than the maximum response.^29^

Indices of arterial stiffness (as measured by PWV^27^) and atherosclerosis (as measured by carotid-intima media thickness load^30^) were also linked to more lacunes and atrophy, despite a lack of co-existent vascular disease or macroscopic evidence of atherosclerosis in our patients. This may indicate that a combination of large vessel disease resulting from smoking and hyperlipidaemia, and small vessel dysfunction, caused by CADASIL, is particularly damaging: alternatively, since there was no macroscopic radiological or clinical evidence of atherosclerosis, these indices may be abnormal as a consequence of CADASIL small vessel arteriopathy. Clearly there are complex interactions between these factors which require larger group numbers to unravel. A recent longitudinal study reported that smoking worsened disease outcomes, but age and hypertension had little effect on disease progression.^12^

CADASIL is characterised by damage to smooth muscle cells.^4^ Reduced CBF and attenuated CVR have been demonstrated to precede histological changes in mouse models.^31^ Whilst models fail to recapitulate human CADASIL entirely, they suggest that smooth muscle cell dysfunction leads to the development of brain lesions. Capillary dysfunction, secondary to loss of pericytes^32^ may also have a role.^3^ In human studies, reduced CBF and cerebral blood volume have been associated with worse clinical or radiological outcomes,^6,33^ and impaired CVR has been associated with disability.^34^ Despite the role of CVR in the development of manifestations of CADASIL remaining unclear, it has already been used as an endpoint in therapeutic trials of oral acetazolamide, which improved CVR and CBF,^35^ and atorvastatin, which did not alter cerebral haemodynamics.^36^ This study provides more evidence that it may have a role in pathophysiology, and hence potential as a biomarker of disease progression.

The role of hypertension in CADASIL remains unclear. We found patients with lower resting systolic BP had lower CVR. A lower BP profile has previously been described in CADASIL due to reduced daytime values,^37^ and MABP was positively correlated to global cognitive impairment. Therefore low BP may either be harmful or reflect more severe disease.^37^ Alterations in BP may be explained by central damage to circadian control, or to autonomic centres. An alternative hypothesis is that tissue in CADASIL has increased capillary transit time heterogeneity (CTTH), and reduced CBF (and perhaps BP) is a protective mechanism to minimise heterogeneity and preserve oxygen extraction.^38^ BP management of these patients needs further investigation. We did not correct for change in BP during reactivity measurements as we did not measure BP in the MRI scanner, and only twice during hypercapnia challenge. Lack of correction for BP fluctuations during hypercapnia could confound CBF and CVR, even if it does not correlate with reactivity measures.

Strengths of this study include a CADASIL population with a wide age range, and a number of affected exons, who lacked conventional risk factors for stroke such as hypertension, diabetes or carotid disease, meaning that cerebral pathology is likely to reflect CADASIL solely. All tests were performed by a single, trained, rater, and attendance at 100% of visits was achieved. High resolution MRI scans were available, with hypercapnic challenge undertaken with monitoring of inspired and expired gases.

There are limitations to our study. The number of subjects is small, largely due to the comprehensive assessment required. Multiple exploratory univariate comparisons may over-estimate the strength of relationships, and adjustment for confounding by variables that are potentially highly correlated in a multivariate model was not possible due to the small sample size. The multimodal analysis of vascular function has not previously been done, and we therefore did not define a single prior hypothesis regarding these relationships. Future studies could use these data to select test panels appropriate to the clinical question. Subjects were asked to refrain from nicotine, caffeine and alcohol, but we did not fast patients or stop any medication. Testing took place at multiple visits over a maximum three-month period, although no subject experienced a new stroke or hospital admission between visits. There was some variation in the time of testing between subjects. We cannot exclude these factors are relevant to our results. Whilst performing all tests on the same day would be ideal, both MRI and cognitive tests are arduous for patients, and completion of protocol procedures would likely have been lower.

Hypercapnia challenge MRI is a challenging technique and CVR was calculable in only 13 patients, predominantly due to inaccurate end-tidal CO_2_ assessment secondary to poor mask seal. Other gas delivery methods are available, and may prove more reliable^39^ but are more expensive and may be less tolerable. Dichotomisation of radiological variables by their median was performed to simply the clinical interpretation^12^ and is arbitrary; however, it is unlikely that the relationships that exist between vascular dysfunction and damage are linear, rather there may be a threshold at which impaired vasoreactivity has a deleterious effect.

The lack of a control group limits interpretation of our results to some extent, but selecting an appropriate control population is challenging. We know symptomatic CADASIL patients differ from age-matched controls, and a control group from this population would be unlikely to address key components of this study to explore the relationships of vasoreactivity and radiological or clinical markers of cerebral small vessel disease, since these features are rarely present in healthy individuals. Patients with sporadic small vessel disease are heterogenous, typically older, and with multiple confounding co-morbidities and medications, and thus present other concerns as a valid control population. Screening control populations for potentially pathogenic mutations in NOTCH3 or other relevant genes presents ethical issues when this is undertaken solely for research, and cannot take into account the potential presence of currently unknown pathogenic mutations. Asymptomatic CADASIL patients diagnosed via genetic screening might provide a useful comparator population, but few asymptomatic patients take up screening in this disease.^40^

Impaired vasoreactivity is associated with increased numbers of lacunes, an important established correlate of clinical severity. Large vessel disease also plays a crucial role. To establish if vasoreactivity could function as a potential biomarker in CADASIL, as well as better understanding its place in pathophysiology longitudinal analysis is required. In light of our finding of low systolic BP being associated with lower CVR, assessment of the role of autonomic dysregulation in CADASIL pathophysiology may be warranted.

## Funding

The author(s) disclosed receipt of the following financial support for the research, authorship, and/or publication of this article: The study was funded by a project grant from the Chief Scientist Office, Scotland (ETM/244) and by a Centre of Research Excellence award from the British Heart Foundation (reference RE/13/5/30177).

## Supplementary Material

Supplementary material
