# Supplementary material for: Vasoreactivity in CADASIL: Comparison to structural MRI and neuropsychology
Source: J Cereb Blood Flow Metab. 2017 May 24;38(6):1085–95. doi: 10.1177/0271678X17710375 (PMC5998994; doi:10.1177/0271678X17710375)
Supplement: Supplementary material [file JCB710375_supplementary_material.pdf]

Supplementary Table 1: Peripheral vascular assessment – methods and background

Vascular measure	Method	Background
Pulse wave analysis (PWA)	Applanation tonometry used to record radial artery pressure waveforms with an automated device (SphygmoCor; AtCor Medical, Sydney, Australia) normalised to heart rate 75bpm. Mean of two readings used.	Assessment of the arterial pressure waveform which can reflect arterial stiffness and left ventricular load. Related to cardiovascular risk and mortality.[1]
Pulse wave velocity (PWV), m/s	Distance between carotid and femoral arteries measured and divided by time delay between carotid and femoral pulses, calculated by ECG-gated applanation tonometry (SphygmoCor; AtCor Medical, Sydney, Australia).	Velocity of the pulse wave along aorto-iliac pathways is the gold-standard measure of arterial stiffness and has predictive value for cardiovascular outcomes.[2]
Reactive hyperaemia index (RHI), %	Digital pulse amplitude in test and control arms recorded during baseline, 5 min occlusion (BP cuff inflated to 200mmHg on the test upper arm), and following cuff deflation. A computerised algorithm (Endo-PAT2000, Itamar Medical Ltd, Caesarea, Israel) calculated the reactive hyperaemia index.	Automated method of evaluating endothelial function in microcirculation of the finger.[3]
Flow mediated dilatation (FMD) of brachial artery, %	Ultrasound imaging of brachial artery at rest, and following 5 min of forearm occlusion at 200mmHg. The maximum peak in diameter post cuff deflation compared to baseline was calculated (flow mediated dilatation, FMD, %) (Brachial Analyzer, MIA LLC, IA, USA).	Measurement of brachial artery dilatation following shear stress created by forearm ischaemia. A non-invasive assessment of endothelial function.[4]
Carotid intima media thickness (CIMT), mm	B-mode ultrasound and a 7MHz probe (Acuson, Sequoia, Siemens Medical Solutions) used to obtain images of the posterior intima media layer in two planes. Automatic border detection software (Siemens Syngo® Arterial Health Package) used to measure the carotid intima media thickness (CIMT) and the average of all readings used.	A marker for early stages of generalised atherosclerosis which is associated with age, sex, blood pressure, BMI, cholesterol and diabetes.[5]

Supplementary Materials.

1. Mancia G, Laurent S, Agabiti-Rosei E, Ambrosioni E, Burnier M, Caulfield MJ *et al.* Reappraisal of European guidelines on hypertension management: a European Society of Hypertension Task Force document. *Journal of Hypertension* 2009; 27(11)**:** 2121-2158.

2. Laurent S, Boutouyrie P, Asmar R, Gautier I, Laloux B, Guize L *et al.* Aortic Stiffness Is an Independent Predictor of All-Cause and Cardiovascular Mortality in Hypertensive Patients. *Hypertension* 2001; 37(5)**:** 1236-1241.

3. Kuvin JT, Patel AR, Sliney KA, Pandian NG, Sheffy J, Schnall RP *et al.* Assessment of peripheral vascular endothelial function with finger arterial pulse wave amplitude. *American Heart Journal* 2003; 146(1)**:** 168-174.

4. Celermajer DS, Sorensen KE, Gooch VM, Miller, Sullivan ID, Lloyd JK *et al.* Non-invasive detection of endothelial dysfunction in children and adults at risk of atherosclerosis. *The Lancet* 1992; 340(8828)**:** 1111-1115.

5. O'Leary DH, Bots ML. Imaging of atherosclerosis: carotid intima-media thickness. *European Heart Journal* 2010; 31(14)**:** 1682-1689.

Supplementary Table 2: Cognitive outcomes compared to vascular and radiological measures.

		Processing speed	Executive function
**Age**	r_s<br>Sig (2 tail)<br>n	-0.253<br>0.268<br>21	0.056<br>0.815<br>20
**SBP**	r_s<br>Sig (2 tail)<br>n	0.103<br>0.657<br>21	-0.023<br>0.925<br>20
**PWA**	r_s<br>Sig (2 tail)<br>n	0.141<br>0.542<br>21	0.223<br>0.345<br>20
**PWV**	r_s<br>Sig (2 tail)<br>n	-0.243<br>0.302<br>20	0.019<br>0.937<br>19
**CIMT**	r_s<br>Sig (2 tail)<br>n	-0.463<br>**0.04**<br>20	-0.266<br>0.271<br>19
**RHI**	r_s<br>Sig (2 tail)<br>n	-0.155<br>0.525<br>19	-0.316<br>0.188<br>19
**FMD**	r_s<br>Sig (2 tail)<br>n	0.343<br>0.164<br>18	-0.064<br>0.808<br>17
**TCD CVR**	r_s<br>Sig (2 tail)<br>n	0.230<br>0.329<br>20	-0.04<br>0.870<br>19
**GM CBF**	r_s<br>Sig (2 tail)<br>n	0.292<br>0.239<br>18	0.324<br>0.267<br>14
**GM CVR**	r_s<br>Sig (2 tail)<br>n	0.500<br>0.082<br>13	0.258<br>0.394<br>13
**SH CVR**	r_s<br>Sig (2 tail)<br>n	0.291<br>0.334<br>13	0.159<br>0.603<br>13
**nSH**	r_s<br>Sig (2 tail)<br>n	0.174<br>0.462<br>20	0.025<br>0.920<br>19
**nLV**	r_s<br>Sig (2 tail)<br>n	0.667<br>**0.001**<br>20	-0.344<br>0.149<br>19
**No of microbleeds**	r_s<br>Sig (2 tail)<br>n	-0.226<br>0.353<br>19	-0.143<br>0.572<br>18
**nBV**	r_s<br>Sig (2 tail)<br>n	0.512<br>**0.021**<br>20	0.441<br>0.059<br>19

Abbreviations: CBF = cerebral blood flow; CVR = cerebrovascular reactivity; PWA = pulse wave analysis (measuring augmentation index at 75 bpm); PWV = pulse wave

velocity; GM = grey matter; CD = transcranial Doppler ultrasound; SH = subcortical hyperintensity; SBP = Systolic blood pressure; nSH = normalised subcortical hyperintensity volume; nLV = normalised lacune volume; nBV = normalised brain volume.

Supplementary Materials.

Supplementary Table 3: Clinical outcomes and scales compared to vascular measures

		History of stroke		Modified Rankin Scale		NIHSS		HADS depression		HADS anxiety	
		No	Yes	0-1	≥2	0	≥1	0-7	≥8	0-7	≥8
**Age**	n	11	11	15	7	17	5	12	9	13	8
	mean (SD)	46 (12)	53 (9)	49 (12)	52 (10)	49 (11)	51 (12)	49 (11)	49 (13)	48 (12)	51 (11)
	p value		0.088*		0.581*		0.446*		0.808*		0.645*
**SBP**	n	11	11	15	7	17	5	12	9	13	8
	mean (SD)	122 (12)	118 (10)	122 (9)	115 (12)	120 (10)	120 (15)	123 (10)	117 (12)	122 (10)	118 (12)
	p value		0.513		0.152		0.964		0.295		0.445
**PWA**	n	11	11	15	7	17	5	12	9	13	8
	mean (SD)	17 (15)	17 (12)	16 (13)	18 (14)	16 (13)	20 (15)	18 (14)	15 (13)	13 (13)	22 (13)
	p value		0.975		0.806		0.543		0.657		0.168
**PWV**	n	11	10	15	6	17	4	12	8	13	7
	mean (SD)	7.3 (1)	8.0 (1.2)	7.6 (0.9)	7.6 (1.5)	7.5 (1.0)	8.3 (1.4)	7.6 (1.0)	7.6 (1.4)	7.5 (1.1)	7.7 (1.3)
	p value		0.188		0.918		0.214		0.956		0.798
**CIMT**	n	11	10	15	6	16	5	12	8	13	7
	mean (SD)	0.63(0.1)	0.65(0.1)	0.62(0.1)	0.69(0.1)	0.64(0.1)	0.66(0.1)	0.61(0.1)	0.67(0.1)	0.61(0.9)	0.67(0.1)
	p value		0.692		0.161		0.669		0.230		0.162
**RHI**	N	10	10	15	5	15	5	12	7	13	6
	mean (SD)	2.2 (0.9)	2.1 (0.5)	2.2 (0.7)	2.0 (0.5)	2.2 (0.7)	2.1 (0.6)	2.1 (0.7)	2.2 (0.8)	2.2 (0.8)	2.0 (0.6)
	p value		0.657		0.583		0.857		0.732		0.665
**FMD**	N	11	7	14	4	15	3	12	6	13	5
	mean (SD)	4.6 (1.9)	3.1 (1.6)	4.1 (2.1)	3.9 (1.1)	4.0 (2)	4.2 (0.6)	4.2 (2.1)	3.6 (1.2)	4.1 (2.1)	4.0 (1.0)
	p value		0.096		0.831		0.876		0.543		0.948
**TCD CVR**	n	10	11	14	7	16	5	11	9	12	8
	mean (SD)	20 (10)	19 (12)	22 (12)	14 (8)	20 (12)	19 (10)	20 (12)	17 (10)	19 (13)	18 (8)
	p value		0.840		0.116		0.905		0.961		0.955
**GM CBF**	n	11	8	15	4	17	2	12	6	13	5
	mean (SD)	51 (12)	51 (6)	50 (10)	54 (8)	50 (10)	57 (7)	51 (9)	51 (11)	50 (10)	54 (7)
	p value		0.904*		0.554		0.384		0.961		0.467
**GM CVR**	n	8	5	11	2	12	1	10	3	11	2
	mean (SD)	12 (7)	6 (12)	11 (9)	-0.9 (4)	8 (8)	28 (NA)	12 (9)	1 (5)	11 (9)	2 (9)
	p value		0.344		0.083		-		0.076		0.254
**SH CVR**	n	8	5	11	2	12	1	10	3	11	2
	mean (SD)	10 (7)	7 (9)	11 (7)	0.9 (1)	8 (7)	23 (NA)	11 (8)	2 (2)	10 (8)	2 (7)
	p value		0.415		0.105		-		0.083		0.342

*Non-parametric test used. NA = not available; - = no statistical test performed.
